# Targeting G-quadruplexes to achieve antiviral activity

**DOI:** 10.1016/j.bmcl.2022.129085

**Published:** 2023-01-01

**Authors:** Emanuela Ruggiero, Sara N. Richter

**Affiliations:** a Department of Molecular Medicine, University of Padua, Italy; b Microbiology and Virology Unit, Padua University Hospital, Padua, Italy

**Keywords:** G-quadruplex, Virus, Antiviral activity, Non-canonical nucleic acids, Ligands, G-quadruplex, G4

## Abstract

With the emergence of new viruses in the human population and the fast mutation rates of existing viruses, new antiviral targets and compounds are needed. Most existing antiviral drugs are active against proteins of a handful of viruses. Most of these proteins in the end affect viral nucleic acid processing, but direct nucleic acid targeting is less represented due to the difficulty of selectively acting at the nucleic acid of interest. Recently, nucleic acids have been shown to fold in structures alternative to the classic double helix and Watson and Crick base-pairing. Among these non-canonical structures, G-quadruplexes (G4s) have attracted interest because of their key biological roles that are being discovered. Molecules able to selectively target G4s have been developed and since G4s have been investigated as targets in several human pathologies, including viral infections. Here, after briefly introducing viruses, G4s and the G4-binding molecules with antiviral properties, we comment on the mechanisms at the base of the antiviral activity reported for G4-binding molecules. Understanding how G4-ligands act in infected cells will possibly help designing and developing next-generation antiviral drugs.

## Graphical abstract

**Figure:**
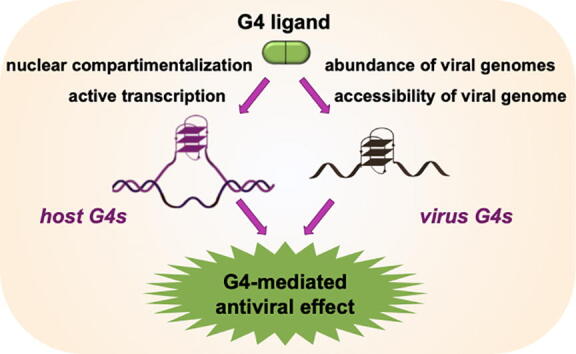

## Introduction

### Virus structure and life cycle

**Fig. 1:**
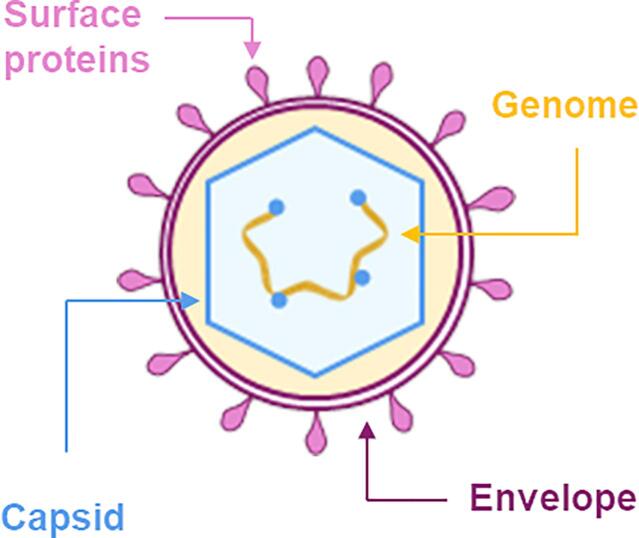
General virus structure with the main viral components shown.

Viruses are microorganisms that must exploit the host organism to replicate. The two basic structural components of viruses are the nucleic acid genome, formed by double- or single-stranded DNA or RNA, and the capsid, a shell made by multiple copies of few viral proteins which protect the viral nucleic acid inside it (Fig. 1).^1^ The capsid also serves for cell-entry, genome uncoating and intracellular trafficking.^2^, ^3^ Some viruses have an additional external layer, the envelope, which is derived from the cell membrane of the infected host and modified with inserted and exposed viral glycoproteins. The viral genome may also encode for proteins that promote gene expression and facilitate replication and assembly of the virus particles; usually the simplest and smallest viruses exploit these functions from the host cell machinery.^4^.

The viral genome is composed of either DNA or RNA in different shapes (circular, linear), arrangements (single- or double-stranded) and numerosity (one copy, two copies or fragmented). Based on their genome type, viruses are classified in seven groups according to the Baltimore classification.^5^.

**Fig. 2:**
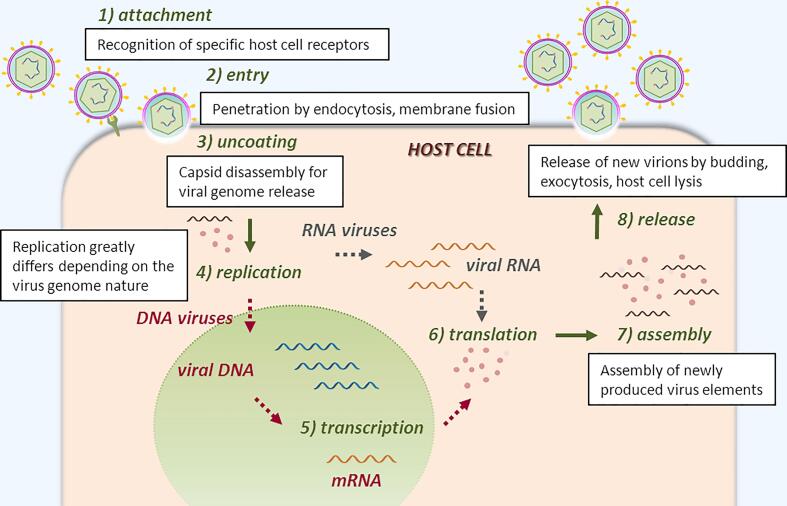
Simplified virus life cycle. 1) Attachment: the virus recognizes and binds to host cell’s surface receptors or proteins to enter the cell. 2) Viral entry: according to different pathway, the viral particle enters the cell. 3) Uncoating: upon penetration, the viral genome is uncoated and released into the host cell’s cytoplasm. 4) The viral genome nature determines the location and the strategy employed for genome replication: most of DNA viruses replicate in the cell nucleus, while the majority of RNA viruses replicate in the cytoplasm. For retroviruses, retrotranscription takes place in the cytoplasm, while integration occurs in the cell nucleus. 5–6) Transcription and translation: viral genes are transcribed to provide the viral proteins necessary for further steps. 7) Assembly: all newly synthesized viral genomes and proteins are assembled to form new virions. 8) Release: the viral progeny is released outside the host cell by several mechanisms so to infect new cells and propagate.

Eight steps are generally necessary for a virus to successfully infect a host cell, produce new infectious viral particles, the virions, and release them ^6^ (Fig. 2): 1) the virus attaches to a host cell (*attachment*) and 2) if viral proteins recognize cell receptors, the virus genetic material is delivered inside the cell (*entry*) and 3) exposed outside the capsid (*uncoating*); 4) during replication the viral DNA or RNA is replicated (*replication*) and 5),6) viral proteins expressed (*transcription, translation*); 7) new virions are assembled from the newly formed viral proteins and nucleic acids (*assembly*) and 8) released outside the host cell (*release*).

### Antiviral drugs

There are 130 viruses reported to infect the human species and cause diseases, which span from benign symptoms that include common cold, respiratory and gastrointestinal manifestations, fever, skin lesions, to severe or fatal outcomes, such as encephalitis, hemorrhagic fever, hepatitis, AIDS, and are accountable for millions of deaths worldwide.^7^.

Hence, development of antiviral drugs has been a major aim since the era of antimicrobial drugs and since the US Food and Drug Administration (FDA) approved the first antiviral drug ‘idoxuridine’ in 1963 to treat the infection caused by herpes simplex virus.^7^ Emergence of the human immunodeficiency retrovirus (HIV) and consequent acquired immune deficiency syndrome (AIDS) epidemic around the globe during 1980s, triggered solid research efforts that led to several antiretroviral drugs becoming available as well as the identification of basic viral molecular mechanisms. Even though successful antiviral drugs have been developed, the intimate interaction of viruses with the host cells has made it difficult to design drugs that are at the same time safe and effective. As a results, to date, about 120 antiviral compounds are available against only 10 human viruses, i.e. HIV, Hepatitis C virus (HCV), Hepatitis B virus (HBV), Influenza virus, Severe Acute Respiratory Syndrome Coronavirus-2 (SARS-CoV-2), Herpes simplex virus (HSV), Human cytomegalovirus (HCMV), Varicella-zoster virus (VZV), Human papillomavirus (HPV) and Respiratory syncytial virus (RSV).^8^.

Most antiviral drugs are small molecules but proteins, peptides and oligonucleotides are also present. Most of them target viral mechanisms that include nucleic acid replication, entry/fusion, uncoating, protease activity, but compounds that target host cell pathways also exist, either in monotherapies or in combination with drugs that target viral components. The antivirals directed against the host are mainly immunomodulatory compounds, such as Interferon proteins in different types and formulations that enhance the immune response and elicit an antiviral state. Dexamethasone, an immunosuppressant and anti-inflammatory drug, is used to avoid the severe respiratory complications of SARS-CoV-2 infected and hospitalized patients.^9^ Some drugs inhibit host cytochrome protein CYP3A which in turn would metabolize and inactivate direct antiviral drugs given in combination. One anti-HPV compound inhibits cell division and two other anti-HIV drugs target cell receptors that are required for virus entry into the cell.

A major challenge in antiviral drug development is resistance, which is a commonly reported issue affecting approved antiviral drugs that directly act against viral targets or virus-host interaction. Drug resistance is particularly common in viruses with RNA-dependent RNA polymerase or retrotranscriptase activity due to the error-prone nature of these enzymes (up to 1 mutated nucleotide over 10^4^ replicated bases), the rapid rate of viral replication (e.g., 10.3x10^9^ and 10^13^ new HIV-1 and HBV virions, respectively, in the human host in 24 h) and frequent recombination events.^10^, ^11^.

Because of the described issues, new antiviral targets in the virus or in the host cell are highly sought to expand the antiviral drug armamentarium in case of resistant virus strains and towards a larger number of viruses.

### Non canonical nucleic acid structures: G-quadruplexes

**Fig. 3:**
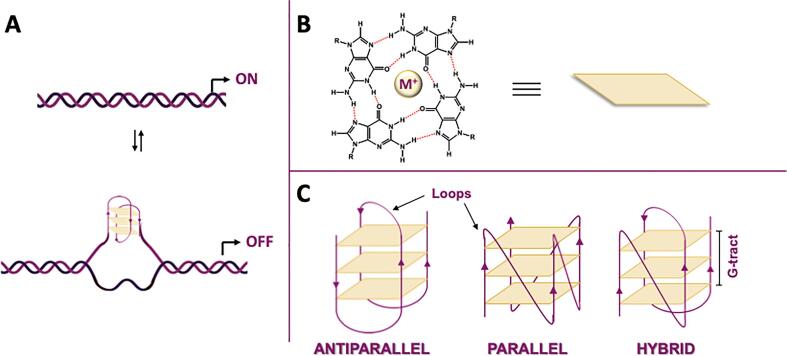
G-quadruplex structure. A) A G4 formed within double-stranded DNA; B) Hoogsteen H-bonds among four guanines which form a G-tetrad; C) Different topologies of intramolecular G4 structures.

Non-canonical nucleic acid structures form when base pairing other than the classic Watson and Crick is involved. Among them, G-quadruplexes (G4s) are structures that form in G-rich sequences of DNA or RNA(Fig. 3A),^12^ when four Gs base pair via Hoogsteen-type hydrogen bonds to yield planar square structures called G-quartets (Fig. 3B). The π-π interactions between the aromatic systems of the G-quartets leads to formation of the G4, when stacking of at least two G-quartets occurs (Fig. 3C). Monovalent cations, usually potassium (K^+^) in the physiological environment, specifically support G4 formation, stability and topology by relieving the repulsion among oxygen atoms that arises in the central cavity. The same sequence can adopt different G4 conformations (Fig. 3C).^13^ Intramolecular (i.e. monomolecular) G4s can form from the following general sequences G_m_X_n_G_m_X_o_G_m_X_p_G_m_, where m is the number of G residues in each G-tract, which are directly involved in G-quartet Hoogsteen interactions. X_n_, X_o_ and X_p_ are the linker or loop sequences connecting the G-rich tracts involved in G4 formation and can be any combination of residues, including G. G4 structures with discontinuities in G-stretches causing bulges have also been reported.^14^ The recent availability of large datasets on G4 formation has enabled the application of machine learning to predict G4 forming propensity.^15^.

Computational as well as deep-sequencing approaches have demonstrated that in the human genome over 700,000 regions exist that could potentially fold into G4 structures.^16^ The physiological relevance of G4 structures is further supported by the existence of proteins that are able to bind and unfold G4s (see G4 Interacting Proteins Database).,^12^, ^17^, ^18^ Mutations and/or deletions of these proteins lead to modulation in G4 formation.^19^ Based on their interaction with proteins and localization in the cell genome, G4s have been implicated in several key biological processes, such as telomere protection and telomerase recruitment,^20^ genomic and epigenetic instability,^21^, ^22^, ^23^ obstacles to the replication machinery,^24^ and triggers of initiation of DNA replication..^25^ G4s are strongly enriched at promoters, especially of oncogenes: more than 40 % of human promoter regions harbor at least one G4 motif.^26^. Recent evidence has pointed to G4s as modulators of transcription, where their formation corresponds to increased transcription levels and interaction with transcription factors.^27^, ^28^, ^29^, ^30^.

### G-quadruplexes in viruses

Besides mammalian cells, G4s have been described also in yeast, plants, bacteria, archea and viruses. Because of the recent SARS-CoV-2 pandemic, viruses in general have rightfully gained much more attention than in the past years. Formation of G4s has now been predicted in all human viruses ^31^ and experimentally shown in all the main virus groups. These have been recently reviewed in 32, 33.^34^ Briefly, G4s have been described in double-stranded DNA viruses: in the *Aphaherpesvirinae* herpes simplex virus types 1 and 2 (HSV-1 and 2) and varicella zoster virus,^35^, ^36^, ^37^, ^38^ in the *Betaherpesvirinae* human cytomegalovirus (CMV)^39^, ^40^ and human herpesvirus 6 (HHV-6),^41^ in the *Gammaherpesvirinae* Epstein-Barr virus (EBV)^42^, ^43^ and Kaposi’s sarcoma herpesvirus (KSHV)^44^, ^45^; in *Papillomaviridae*^46^ and *Adenoviridae*.^47^ In viruses with single-strand RNA genome with positive polarity, G4s have been found in *Coronaviridae*, including SARS-CoV-2,^48^ *Flaviviridae* as hepatitis C virus (HCV)^49^ and those transmitted by mosquitos, such as Zika virus (ZIKV)^50^, ^51^ and West Nile virus (WNV).^52^ In the same group, Chikungunya virus (CHIKV)^53^ belonging to *Togaviridae* and rhinovirus (RhV)^54^ belonging to *Picornaviridae* were shown to present G4s. In viruses with single-stranded RNA genome with negative polarity, G4s have been reported in Ebola (EBOV)^55^ and Marburg (MARV)^56^ viruses belonging to *Filoviridae* and in Influenza virus belonging to *Orthomyxoviridae*.^57^, ^58^ Finally, G4s have been described in two viruses that present reverse transcriptase activity, i.e. the human immunodeficiency virus (HIV) belonging to *Retroviridae*,^59^, ^60^, ^61^, ^62^, ^63^, ^64^ where also animal viruses have been shown to display the same G4 arrangement,^65^, ^66^ and in the hepatitis B virus,^67^ belonging to *Hepadnaviridae*.

The viral G4s have been shown to modulate virus replication and transcription in most cases, to affect translation and integration of the viral genome in the host chromosome,^41^, ^48^ modulate antigen presentation,^43^ be involved in virus compartmentalization into vesicles.^68^ In some cases, viral proteins able to specifically recognize viral and host G4s have been reported;^36^, ^69^, ^70^, ^71^ in other cases, cell proteins that interact with viral G4s and affect viral replication have been described.^72^, ^73^, ^74^

### G-quadruplex ligands

Because of the relevance of G4s in cell biology, many G4 ligands have been developed to date. Most of them display an aromatic surface that stacks with the external G-tetrads, one positive charge or basic groups that bind to the G4 grooves or loops, and steric hindrance to avoid intercalation with the double-stranded DNA.^75^ Even though these G4 ligands lack traditional ‘drug-like’ properties, one of them has shown significant accumulation and efficacy in tumor xenografts of human cancers.^76^ Some compounds have reduced planarity while maintaining G4 binding due to their interaction with the groove and backbone phosphates.

**Fig. 4:**
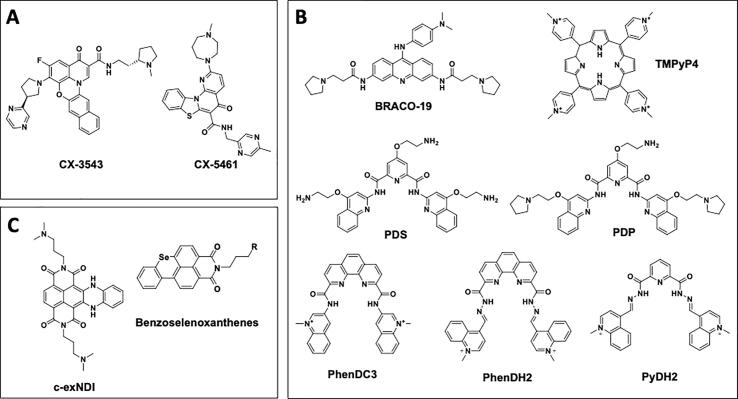
Chemical structures of the discussed G4 ligands.

To date, around 2800 small molecules targeting G4 structures have been reported (see G-Quadruplex Ligands Database 2.2 https://www.g4ldb.com/).^77^ Two of these ligands have entered clinical trials (Fig. 4A). CX-3543, also named quarfloxin, is a fluoroquinolone that disrupts the binding between ribosomal G4s and nucleolin in the nucleolus, thus inhibiting ribosome biogenesis.^78^ Although CX-3543 advanced to phase I clinical trials as a candidate therapeutic agent against several tumors, it lacked sufficient efficacy to warrant further clinical development. Another fluoroquinolone, CX-5461, has been recently proved to inhibit topoisomerase II in G4-containing regions, by selectively binding to G4s.^79^ Both CX-3543 and CX-5461 bind and stabilize a broad spectrum of G4 structures, including those at oncogene promoters, e.g. *c-MYC*, *c-KIT*, and telomeres.^80^ From 2016, CX-5461 is in phase I clinical trials for patients with *BRCA1/2* deficient tumors, constituting the most advanced G4 ligand in the clinics at the moment.^81^.

G4 ligands tested against viruses include BRACO-19, TMPyP4, Pyridostatin and its derivative PDP, PhenDC and its derivatives, and quindoline derivatives (Fig. 4B). These, albeit interacting with both cell and viral G4s, have been shown to exert antiviral activity,^32^, ^34^, ^38^ In some instances, G4 ligands that targets the viral G4s with a certain degree of selectivity have also been developed: ThT-NE, which serves as an indicator of HCV G4s,^82^ and more proper antiviral agents such as the naphthalene diimide derivative c-exNDI,^83^ gamma-PNAs ^52^ and benzoselenoxanthenes ^84^ (Fig. 4C).

### Proposed antiviral modes of action of G-quadruplex-binding compounds

Because of the less than perfect specificity of the employed G4 ligands to the viral G4s, how comes that good antiviral activities have been observed?

**Fig. 5:**
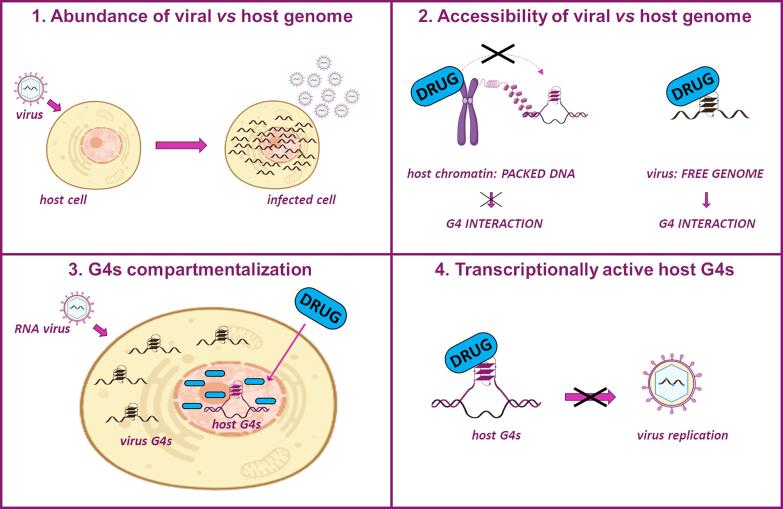
Proposed G4-mediated antiviral mechanisms. 1) The abundance of viral vs host genome, occurring upon virus infection, drives G4-ligands towards viral G4s with respect to the cellular ones. 2) While the host genome is present inside the cell nucleus in a highly condensed (chromatin) form, the viral genome is more accessible to G4 ligands, especially right after being newly synthesized. 3) Since viruses exploit the host transcription machinery to replicate their genome and propagate, targeting transcriptionally active host G4s might indirectly impair virus replication, thus providing a general antiviral effect. 4) When viral replication occurs within the cytoplasm, antiviral G4 ligands that accumulate in the cell nucleus most likely exert their activity trough modulation of host G4s. The blue “DRUG” label indicates a generic G4 ligand.

Different aspects can be evoked to explain the observed effect (Fig. 5):

1) when a virus infects the host cells, it produces many genomes that are then assembled into new virions to be released from the cell. The number of virions released from the cell is called burst size and comprises both infectious and non-infectious virions. It has been estimated that thousands of new virions are formed and released in a short time within a single eukaryotic cell.^85^, ^86^.

The number of sequences actually folded in G4 in human cells at any given moment is about 10000.^87^ In the HSV-1 genome, G4s may form both in about 1,000 bases at repeats 31, 88 and in the promoters of immediate early genes, i.e. genes that control the viral life cycle.^37^, ^36^ There has been no count of the G4s that are actually folded in the HSV-1 genome yet, but considering that the typical HSV-1 burst size is about 1,000 virions per infected cell 89, 90 and having observed that the G4 signal measured by immunofluorescence highly increased upon cell infection and followed the viral genome movement in the host cell,^35^ we can safely assume that the number of viral G4s largely exceeds that of host cell G4s, at least in the highly replicating HSV-1 genome. The same types of considerations can be applied to other viruses, especially those with high G/C rich content genomes and fast replications rates.

2) The replicating and newly formed viral genomes are typically devoid of interacting and protecting proteins that vice versa are present in the host cell genome, with its heavy chromatinization. Thus, even the genome of double-stranded viruses during replication is more exposed and more prone to G4 formation. Consequently, when a general G4 ligand is employed, assuming that the affinity towards the different G4 conformations is similar, the antiviral effects can be likely ascribed to the increased viral G4 targets being available upon virus infection and replication in the host cell, paralleled by the lower availability of the ligand for the host cell G4s.

3) A third mechanism can be proposed: all viruses need to exploit the host cell synthetic machinery for their own replication. The simplest viruses heavily rely on the host cell, the more complex viruses synthesize several own enzymes but still need elements of the host cell. Many human viruses achieve their fast replication by manipulating the host cell cycle, subverting it to their advantage by interfering with specific steps of the cycle.^91^ For instance, some viruses induce quiescent cells to enter the cell cycle or G1-to-S phase transition to increase the available pools of deoxynucleotides and replicate their genomes concomitantly with the synthesis of cellular chromosomes, others arrest the progression from the G2 phase, a period of rapid cell growth and protein synthesis, to the M phase during which cells divide.^92^.

G4-forming sequences are prevalent in genes that trigger the cell cycle ^93^ and G4 folding at gene promoters has been associated with gene transcription.^27^, ^28^ Thus, an increase in host cell G4s can be expected upon virus infection. The use of G4 ligands could block those genes that are transcriptionally modulated, rendering the cell less permissive to viral infection and, in the end, exerting indirect antiviral activity. For instance, HSV-1 has been reported to modulate the amount of MYC,^94^, ^95^ the promoter of which contains G4s that are normally targeted by G4 ligands. In other instances, G4s may be linked to cell receptors that mediate virus entry, as recently shown in SARS-CoV-2 infection.^48^.

4) A final aspect to be considered is the cell compartment of the G4 target. In RNA viruses, for instance, the G4s are produced and maintained in the cytoplasm, therefore G4 ligands that concentrate in the nucleus would not be effective or could exert indirect antiviral activity as proposed above. In light of this, some reported antiviral activities may be rather ascribed to indirect activity on host cell G4s, especially when a virus, such as RNA viruses, replicates in the cytoplasm while the G4 ligand concentrates in the cell nucleus.

The four proposed mechanisms are not exclusive and can be present at the same time, thus further amplifying the antiviral effects of G4 ligands.

## Conclusion and outlook

Considering the emergence of new viruses, the fast mutation rates of the existing ones and the lack of general antiviral drugs, new targets that can be exploited for antiviral purposes are highly sought. G4s could be resourceful elements to be evaluated to this end, since effective antiviral activity can be obtained also when a molecule is not tailored against one single G4. *In vivo* and clinical data will be needed to assess whether G4-binding compounds can effectively be developed into next-generation antiviral drugs.

## Declaration of Competing Interest

The authors declare that they have no known competing financial interests or personal relationships that could have appeared to influence the work reported in this paper.

## Data Availability

No data was used for the research described in the article.
